# Management of recurrent posterior dislocation of a stock temporomandibular joint prosthesis using class II elastic traction: A case report and literature review

**DOI:** 10.1097/MD.0000000000050718

**Published:** 2026-10-02

**Authors:** Liwei Huang, Haoning Li, En Luo

**Affiliations:** a State Key Laboratory of Oral Diseases and National Center for Stomatology and National Clinical Research Center for Oral Diseases, West China Hospital of Stomatology, Sichuan University, Chengdu, Sichuan, China.

**Keywords:** alloplastic reconstruction, condylar resorption, intermaxillary fixation, occlusal instability, orthognathic surgery

## Abstract

**Rationale:**

Dislocation of the mandibular component after total temporomandibular joint replacement (TMJR) is uncommon but can be difficult to manage because manual reduction may fail, and recurrence can occur.

**Patient concerns:**

A 25-year-old woman with bilateral condylar resorption, mandibular retrognathia, class II malocclusion, and severe obstructive sleep apnea underwent bilateral stock TMJR and concomitant orthognathic surgery.

**Diagnoses:**

Posterior dislocation of the left mandibular component occurred intraoperatively and was manually reduced. Posterior dislocation recurred on postoperative day 1 and again on postoperative day 37.

**Interventions:**

After unsuccessful manual reduction, revision surgery was performed to reposition the left mandibular component. Following the recurrence on postoperative day 37, continuous class II elastic traction was applied for 22 days.

**Outcomes:**

Class II elastic traction restored satisfactory occlusion and mandibular position without the need for further surgery. At the 6-month follow-up, the patient maintained stable occlusion and facial profile, a maximum mouth opening of 35 mm, and stable condyle-fossa prosthesis positioning on imaging. The literature review identified 10 publications involving 57 patients with perioperative prosthesis dislocation; 64.3% of the dislocations were detected intraoperatively and 35.7% postoperatively.

**Lessons:**

Recurrent dislocation may occur after TMJR, particularly following extensive mandibular repositioning and postoperative muscular instability. Prompt recognition, appropriate prosthesis repositioning, and postoperative elastic traction may reduce the risk of recurrence and avoid additional surgery.

## 1. Introduction

Temporomandibular joint replacement (TMJR) is used to treat severe temporomandibular joint (TMJ) diseases or injuries, including severe arthritis, joint deformities or developmental abnormalities, advanced degenerative changes, TMJ ankylosis, TMJ tumors, failed TMJ reconstruction, and post-traumatic joint disease.^[1]^ Severe trauma to the TMJ or mandible, particularly condylar fractures, may result in malocclusion, ankylosis, and degenerative changes and, in advanced cases, necessitate TMJR.^[2]^ TMJR is a preferred reconstructive option for skeletally mature patients because it enables immediate restoration of joint function, avoids a 2nd surgical site, corrects skeletal deformity and malocclusion, and improves the predictability of treatment outcomes.^[3]^ Both custom and stock prostheses are widely used in joint reconstruction. Custom prostheses, such as the TMJ Concepts system (TMJ Concepts), are designed to conform more closely to the bone surface. Stock prostheses, including the Biomet Microfixation TMJ Replacement System (Biomet Microfixation Inc.) and the Christensen alloplastic joint system (TMJ Implants Inc.), can be used across a broader range of clinical settings.^[4,5]^ In the present case, the stock Biomet Microfixation TMJ Replacement System was used.

The Biomet stock prosthesis has been reported to have a revision rate of approximately 2.5%, with reasons for revision including infection, heterotopic bone formation, material sensitivity, dislocation, and pain.^[6,7]^ Once the mandibular component dislocates from the fossa component, manual reduction may be difficult because of the rigidity of the prosthetic components and may cause severe pain, screw loosening, or even screw fracture. Such dislocation may necessitate revision surgery and may recur despite component repositioning.^[8]^

Temporomandibular joint prosthesis (mandibular component) dislocation is an uncommon complication, with a reported perioperative incidence of approximately 8%. With timely recognition and appropriate management, the rate of clinically significant postoperative dislocation may be reduced to <1%. Coronoidectomy is currently considered a risk factor for prosthesis dislocation. Detachment of the temporalis muscle from the mandible may contribute to instability, while inflammatory arthropathy and significant elongation of the mandibular ramus during surgery may also increase the risk of joint instability.^[8]^

This report describes recurrent dislocation after TMJR and reviews the available literature to summarize its management and associated risk-control strategies.

## 2. Case report

A 25-year-old woman presented with stertorous breathing and chronic spontaneous TMJ pain. Clinical examination revealed class II malocclusion and anterior dental protrusion. Computed tomography (CT) imaging demonstrated bilateral condylar resorption, loss of the condylar heads, and marked shortening of both mandibular rami. She was diagnosed with bilateral condylar resorption, mandibular retrognathia, and severe obstructive sleep apnea. After preoperative orthodontic alignment and dental decompensation, the patient was admitted for surgery (Fig. S1, Supplemental Digital Content 1).

### 2.1. Surgical procedure

Surgery was performed under general anesthesia with nasotracheal intubation. Preauricular and submandibular incisions were made, followed by bilateral condylectomy. The fossa prosthesis was fixed to the root of the zygomatic arch with titanium screws, while the condyle prosthesis was secured to the lateral aspect of the mandibular ramus, with the joint head properly positioned within the articular fossa prosthesis. After removal of the occlusal splint, mandibular mobility and joint function were assessed under partial muscle activation, and no abnormalities were detected. Bilateral drains were then placed, and the wounds were closed in layers. During the Le Fort I downfracture of the maxilla, an immediate posterior dislocation of the left mandibular component occurred. The dislocation was immediately recognized and manually reduced. Subsequently, a routine Le Fort I osteotomy was completed using the final occlusal splint as a guide.

### 2.2. Postoperative period after the 1st surgery

The patient was transferred to the postanesthesia care unit for recovery and then to the intensive care unit for observation. On postoperative day 1, intraoral examination revealed occlusal derangement, mandibular retrusion, and deviation to the left. CT imaging showed that the titanium screws of the fossa component remained in place, whereas the left mandibular component was displaced posteriorly (Fig. S2, Supplemental Digital Content 2). After manual reduction was unsuccessful, revision surgery was performed under general anesthesia to reposition the left mandibular component.

Following reassessment of bilateral joint mobility and function under partial muscle activation, with no abnormalities detected, left-sided drains were placed, and the wound was closed in layers.

### 2.3. Postoperative period after the 2nd surgery

The patient’s occlusion was satisfactory after the 2nd surgery. The drains were removed on postoperative day 2, and intermaxillary fixation (IMF) was initiated on postoperative day 3. The patient was discharged on postoperative day 6 (Fig. S3, Supplemental Digital Content 3).

### 2.4. Postoperative follow-up

The patient underwent 2 weeks of IMF after discharge and returned to our department on postoperative day 22 for IMF removal. At that time, the occlusion was satisfactory. The patient was advised to perform rehabilitation exercises and attend regular follow-up visits. She reported no discomfort during this period.

On postoperative day 37, the patient returned with mandibular retrusion and deviation to the left. Imaging confirmed recurrent posterior dislocation of the left mandibular component (Fig. S4, Supplemental Digital Content 4). An attempt at manual reduction was discontinued because of intolerable pain.

Class II elastic traction was initiated using two 1/8-inch elastics. At follow-up on postoperative day 45, the patient demonstrated satisfactory occlusion and facial profile. One elastic was subsequently removed, and traction was continued with the remaining elastic. Elastic traction was discontinued on postoperative day 59 (Fig. 1).

**Figure 1.**
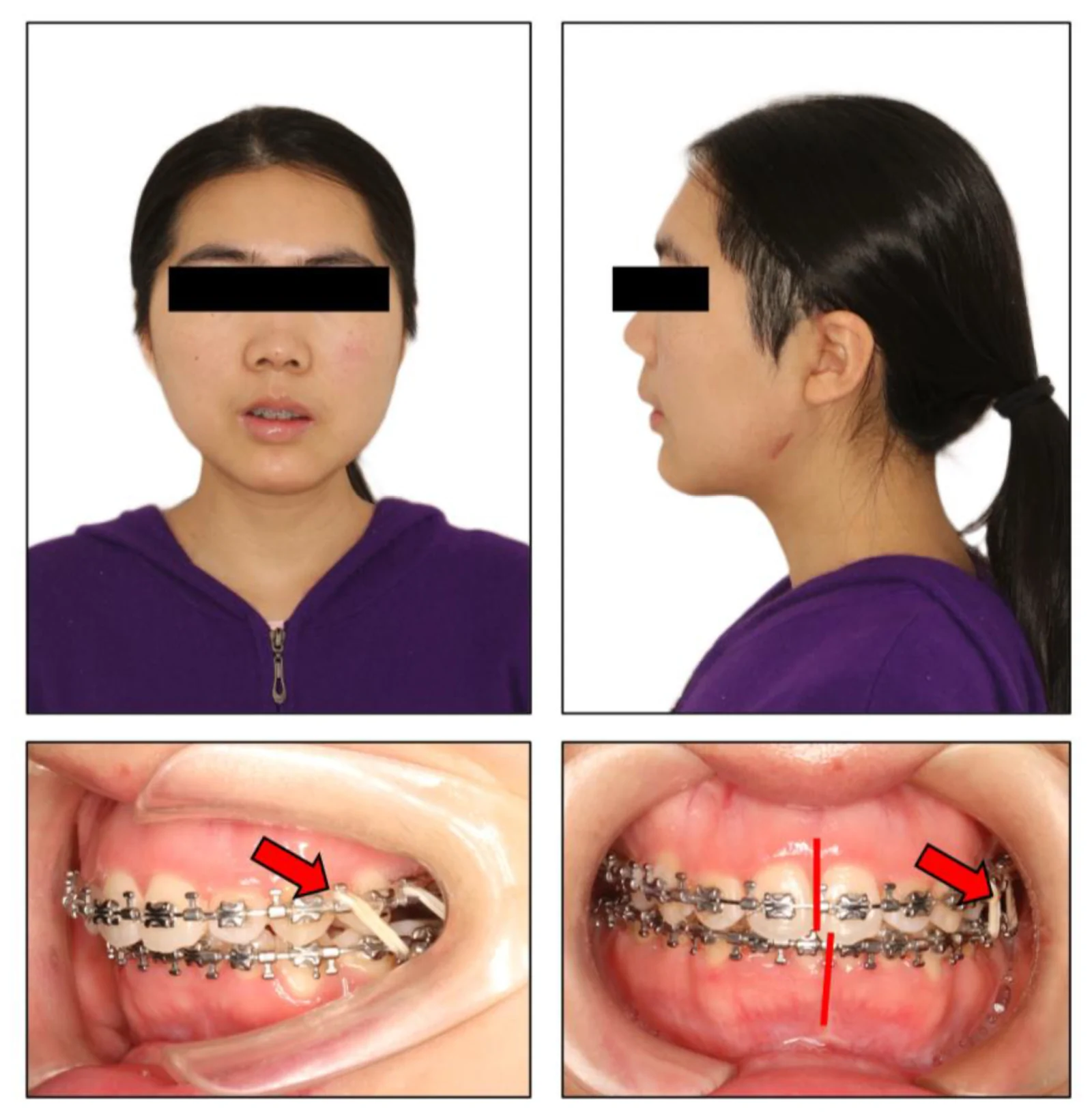
Facial and intraoral photographs of class II elastic traction. The red arrows indicate the class II elastics applied on the left side. The red lines indicate the maxillary and mandibular dental midlines, showing leftward deviation of the mandibular midline.

At the 6-month follow-up, the patient maintained stable occlusion and facial profile, with a maximum mouth opening of 35 mm (Fig. 2).

**Figure 2.**
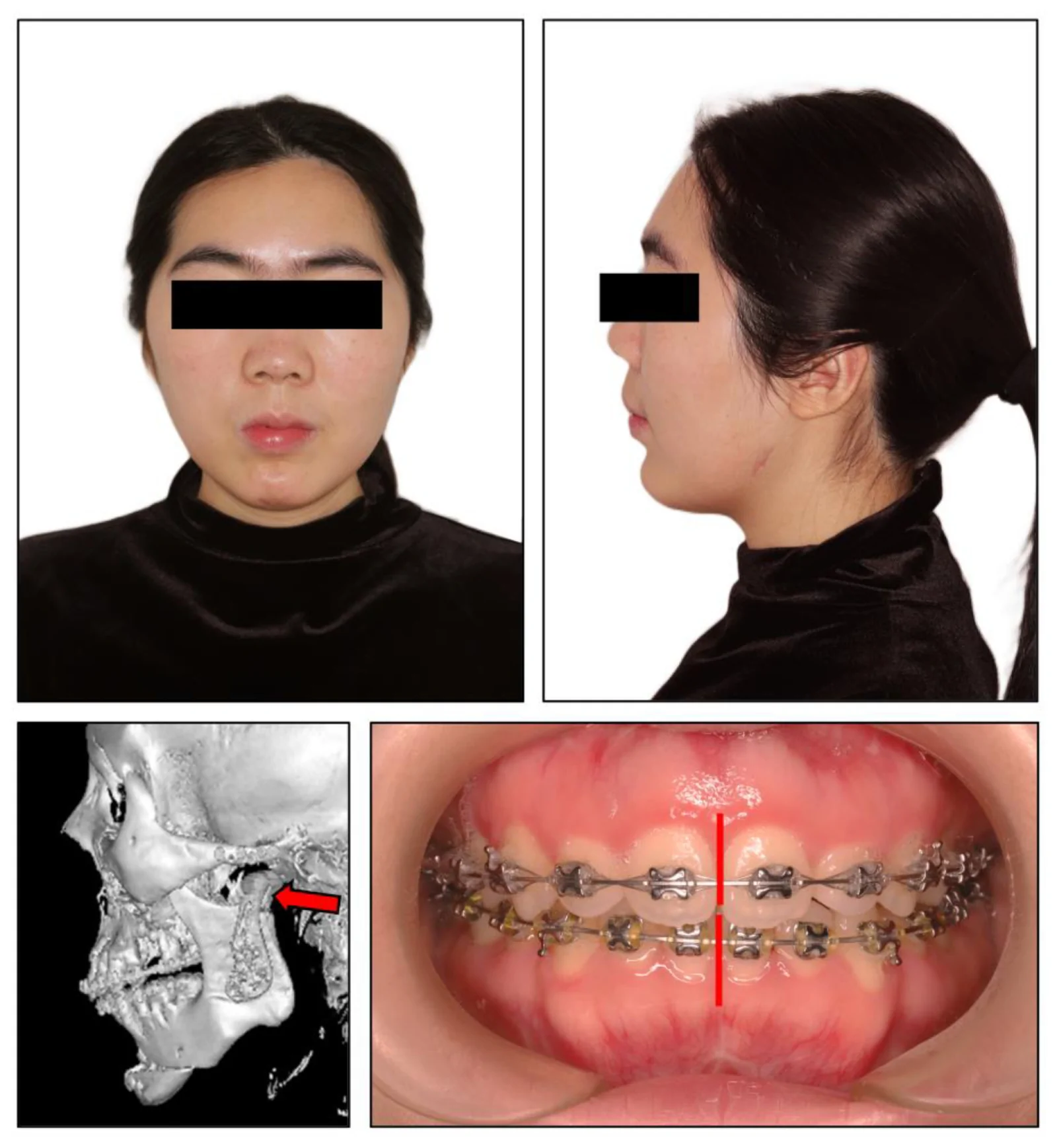
Facial photographs, 3-dimensional CT reconstruction, and an intraoral photograph at the 6-month follow-up. The red arrow indicates the repositioned joint prosthesis. The red lines indicate the approximately aligned maxillary and mandibular dental midlines. CT = computed tomography.

## 3. Literature review

A literature search was conducted in PubMed on March 15, 2025, to identify relevant English-language publications using the search terms: “total joint replacement” [MeSH] AND “joint dislocation” [MeSH]. A total of 10 publications were identified, reporting 57 patients with perioperative joint prosthesis dislocation. To our knowledge, the present case represents the only reported instance of recurrent dislocation.

The 57 patients were reported across 6 countries or regions. The reported dislocations involved 3 widely used TMJ prosthesis systems: TMJ Concepts, Zimmer Biomet, and Christensen. The reported dislocation rates were 10% for the TMJ Concepts system, 3.7% for the Zimmer Biomet system, and 4% for the Christensen system. Dislocations identified and corrected intraoperatively accounted for 64.3% of cases. In such instances, manual reduction was typically followed by elastic traction. Postoperative dislocations accounted for 35.7% of cases. Reported management strategies included manual reduction followed by elastic traction, reduction under general anesthesia, botulinum toxin injection into the masticatory muscles before reduction, and revision surgery to adjust prosthetic positioning.

Additionally, mouth-opening exercises were delayed after reduction to prevent recurrence. No recurrent dislocations were reported after treatment (Table 1).

**Table 1:** Summary of all identified published reports of temporomandibular joint prosthesis dislocation following total temporomandibular joint replacement.

Author	Year	Region	Type of prosthesis	Occurrence (N; %)	Diagnosis	Time	Measurement
Amarista FJ^[6]^	2020	–	TMJ Concepts (custom) Zimmer Biomet (stock/custom) Christensen system (stock)	21 (0.4)	–	–	✓Revision ✓Replacement
Machon V^[9]^	2020	Czech Republic	Zimmer Biomet (stock)	1 (1.6)	–	Post-op	✓Elastic traction
O’Connor RC^[10]^	2016	UK	TMJ Concepts (Custom)	5 (19.2)	–	Intra-op (80) Post-op (20)	✓Immediate relocation ✓Elastic traction
Gonzalez-Perez LM^[11]^	2016	Spain	Zimmer Biomet (stock)	1 (1.8)	–	Post-op	✓Elastic traction
Gruber EA^[12]^	2014	UK	TMJ Concepts (custom)	2 (3.4)	➢ Open bite	Post-op	✓Relocation under general anesthesia ✓Elastic traction
Mustafa el M^[8]^	2013	UK	TMJ Concepts (custom)	11 (10.5)	➢ Degenerative disease ➢ Inflammatory arthropathy ➢ Ankylosis ➢ Costochondral resorption	Intra-op (91) Post-op (9)	✓Elastic traction ✓Late mouth exercises ✓Botulinum toxin A injection
Sidebottom AJ^[13]^	2013	UK	TMJ Concepts (custom)	5 (6.7)	➢ Ankylosis ➢ Open bite	Intra-op (80) Post-op (20)	✓Elastic traction
Murdoch B^[14]^	2013	New Zealand	TMJ Concepts (custom)	1 (2.4)	-	Post-op	✓Delayed physical therapy ✓Elastic traction
Jones RH^[15]^	2011	Australia	TMJ Concepts (custom) Zimmer Biomet (stock)	2 (28.6)	➢ Condylar fracture ➢ Recurrent keratocyst	Post-op	✓Relocation under general anesthesia ✓Elastic traction
Saeed N^[16]^	2002	USA	Christensen system (stock)	2 (4.0)	–	–	–

Intra-op = intraoperative, Post-op = postoperative, TMJ = temporomandibular joint.

Notably, the patient in the present case did not undergo coronoidectomy. Despite prosthesis repositioning through revision surgery, dislocation recurred. This raises an important question: could immediate postoperative elastic traction after the intraoperative dislocation have prevented the early postoperative recurrence and avoided revision surgery? This possibility highlights the potential value of noninvasive elastic traction as both a preventive and therapeutic measure and warrants further investigation.

## 4. Discussion

Alloplastic TMJR can provide stable postoperative occlusion and facial profile. Dislocation is an uncommon but reported perioperative complication. At our institution, Biomet stock prostheses are commonly used for joint reconstruction in end-stage temporomandibular joint disease. The posterior edge of the fossa component is relatively weak, making it prone to anterior or posterior dislocation when the mandibular component exhibits abnormal movement or is subjected to continuous posterior muscle traction. Once the mandibular component dislocates, manual repositioning becomes difficult because of the rigidity of the prosthetic components. Furthermore, recurrent dislocation cannot be completely excluded even after intraoperative repositioning of the mandibular and fossa components, sometimes necessitating multiple surgeries.

Serial digital reconstructions were generated from imaging obtained at different time points to model the patient’s maxilla, mandible, and mandibular component. The existing digital models of the fossa were registered to the skull based on the positions of the titanium screws. Because CT imaging was not available before the 1st dislocation, the dislocated mandibular component was registered to the skull only after final occlusal stabilization, thereby providing the 1st postoperative reconstruction dataset.

After the 1st posterior dislocation, the left mandibular component was completely displaced posterior to the fossa component. During the 2nd surgery, the fossa component was moved slightly posteriorly, while the mandibular component was advanced slightly, centering the prosthetic head within the fossa component.

During the 2nd dislocation, the mandibular component was partially dislocated but remained within the fossa. Class II elastic traction using rubber bands was applied continuously for 22 days, resulting in satisfactory occlusion at approximately 6 weeks postoperatively. Stable condyle-fossa component positioning was confirmed by imaging at the 6-month follow-up (Fig. S5, Supplemental Digital Content 5).

Consistent with previous reports, most perioperative TMJ prosthesis dislocations can be managed by manual reduction followed by elastic traction. However, the present case differed in that recurrent posterior dislocation occurred despite intraoperative reduction and subsequent revision surgery. This difference may be related to the substantial sagittal and vertical repositioning of the mandible, postoperative muscular imbalance, and insufficient posterior restraint provided by the stock fossa component. The successful use of class II elastic traction in this patient suggests that controlled postoperative traction may help stabilize the mandibular component while periarticular soft-tissue adaptation occurs. Nevertheless, this interpretation is based on a single case and requires validation in larger clinical series.

For patients with bilateral condylar resorption and skeletal class II deformity, joint stability may be compromised by capsular laxity and insufficient support from the masticatory muscles. In addition, substantial mandibular rotation during TMJR, particularly when the ramus height is increased, may alter the orientation and force balance of the masseter and temporalis muscles. If the posterior edge of the fossa component cannot provide sufficient retention, posterior or lateral dislocation of the mandibular component may occur. Class II elastic traction appeared sufficient to counteract the posterior and inferior forces acting on the left mandibular prosthesis. Therefore, in patients undergoing TMJR with substantial sagittal or vertical mandibular repositioning, postoperative elastic traction may be considered for an individualized duration to stabilize the occlusion while periarticular soft-tissue adaptation and scar formation occur.^[15]^

Up to 30% of prosthetic dislocations have been reported in patients undergoing or with a history of coronoidectomy.^[8]^ In addition to patient-related factors, intraoperative procedures can contribute to prosthesis dislocation. During the plating of the Le Fort I, especially when excessive rotational opening or vertical pressure is applied to the mandible during downfracture and mobilization of the maxilla, anterior dislocation may occur. Additionally, when seating the maxillomandibular complex to check for bony interferences or during the plating of the Le Fort, posterior dislocation of the joints can occur. This dislocation may not be immediately recognized during plating but may become evident once the IMF is released or on postoperative imaging. Our literature review showed that 64.3% of dislocations were identified intraoperatively. Because some intraoperative dislocations may go unrecognized, their true incidence may be higher. Accordingly, the position of both mandibular components should be reassessed intraoperatively, particularly after closure of the joint wounds and before completion of surgery.

The literature review indicated that the reported dislocation rate of TMJ Concepts prostheses was higher than that of Zimmer Biomet stock prostheses. However, this discrepancy may be influenced by publication bias. The posterior dislocation was attributed to the relatively weak posterior edge of the fossa prosthesis. This weakness is likely related to inherent design characteristics of the Biomet stock prosthesis, which has a less prominent posterior stop than custom-made TMJ prostheses such as the TMJ Concepts system. However, there is currently no clear evidence demonstrating that surgical placement techniques directly cause prosthesis dislocation. Although technical factors during surgery, including maxillary downfracture and mandibular repositioning, may theoretically affect joint stability, their direct role in prosthesis dislocation remains unproven.

Postoperative elastic traction during the 1st week may be beneficial, particularly after an intraoperative dislocation, to reduce the risk of early recurrence. This period may permit early soft-tissue reattachment and scar formation around the mandibular border and angle, reducing the likelihood of dislocation during the period of impaired muscular control. This consideration may be particularly relevant when simultaneous coronoidectomy requires extensive detachment of muscular attachments from the mandibular ramus. Accordingly, the potential need for early postoperative elastic traction should be considered during treatment planning.

## Acknowledgments

This study was supported by the Key Research and Development Support Program of Chengdu (2025-YF09-00015-SN), the Medical Research Project of Sichuan Province (S20250057), the Sichuan University Experimental Technology Research Project (SCU2025051), and the Clinical New Technology Program of West China Hospital of Stomatology, Sichuan University (WCHS-APP-2025-0087).

## Author contributions

**Data curation:** Liwei Huang, Haoning Li.

**Formal analysis:** Haoning Li.

**Investigation:** Liwei Huang.

**Project administration:** En Luo.










