# Supplementary material for: Management of recurrent posterior dislocation of a stock temporomandibular joint prosthesis using class II elastic traction: A case report and literature review
Source: Medicine (Baltimore). 2026 Oct 2;105(40):e50718. doi: 10.1097/MD.0000000000050718 (PMC13633035; doi:10.1097/MD.0000000000050718)
Supplement: Supplementary file 1 [file medi-105-e50718-s001.pdf]

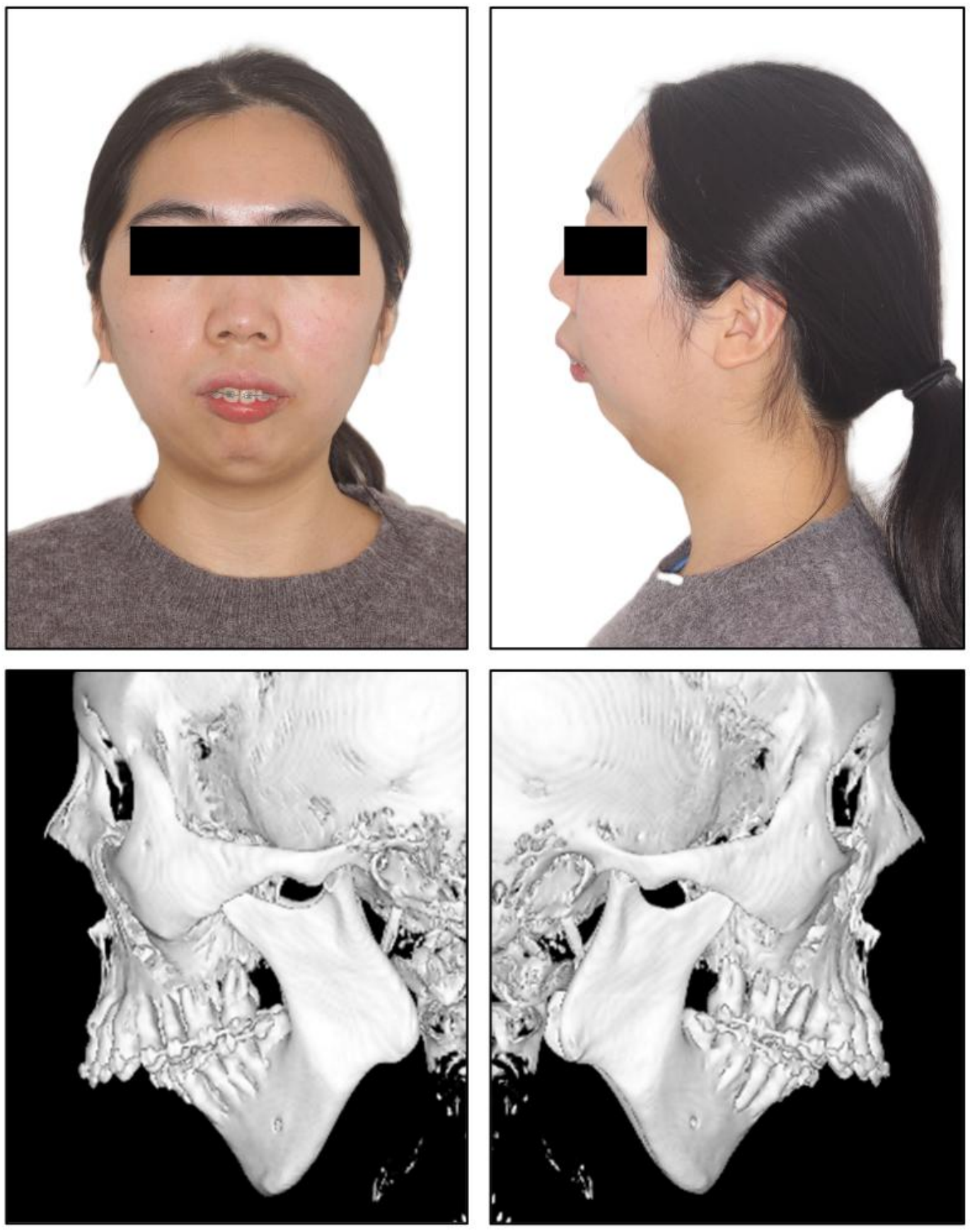

Supplementary figure 1. Preoperative facial and CT image of the patient.

CT imaging demonstrates bilateral shortening of the mandibular rami and mandibular retrognathia.
