# Supplementary material for: Management of recurrent posterior dislocation of a stock temporomandibular joint prosthesis using class II elastic traction: A case report and literature review
Source: Medicine (Baltimore). 2026 Oct 2;105(40):e50718. doi: 10.1097/MD.0000000000050718 (PMC13633035; doi:10.1097/MD.0000000000050718)
Supplement: Supplementary file 3 [file medi-105-e50718-s003.pdf]

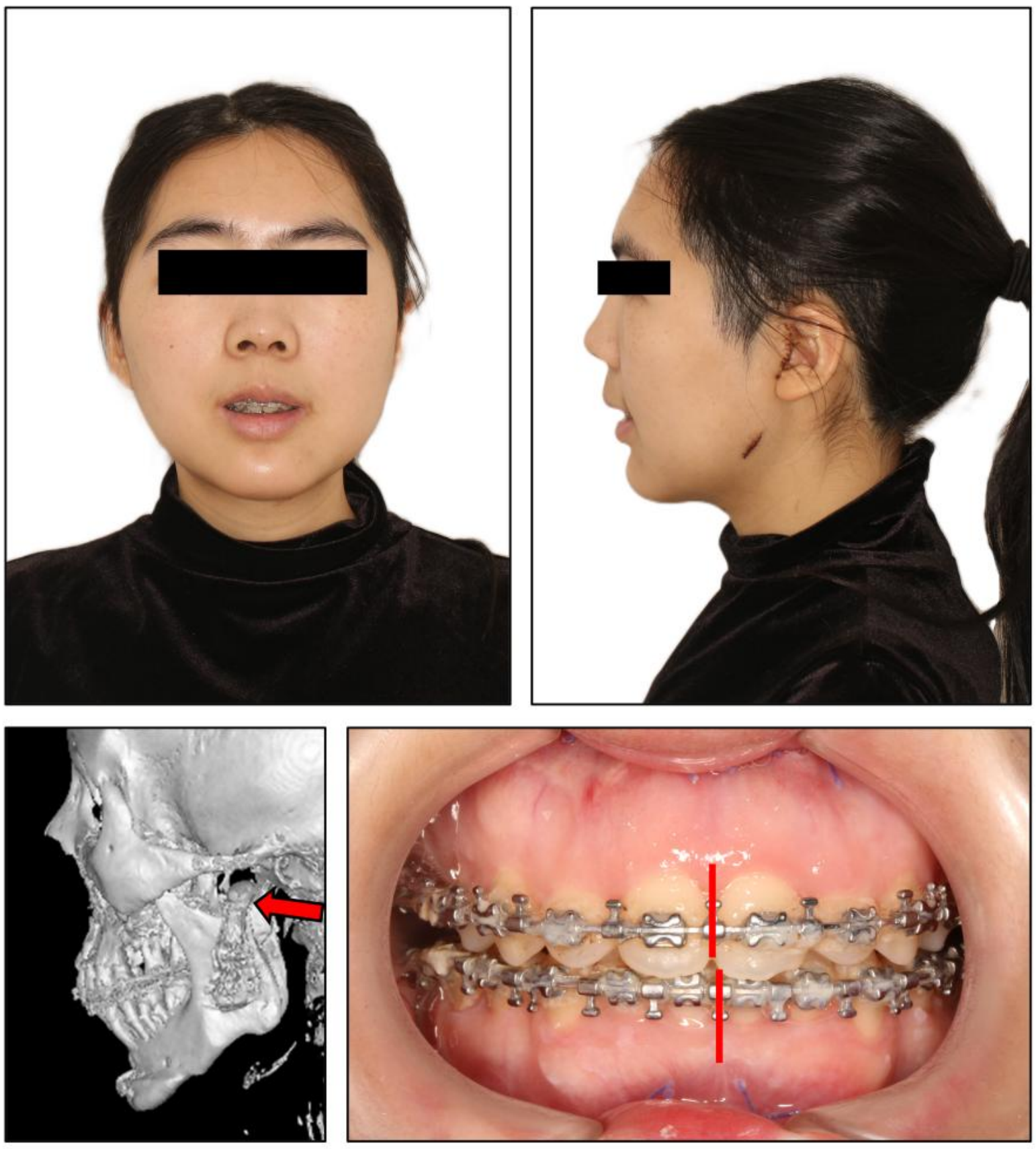

Supplementary figure 3. Facial/CT images and intraoral photograph after second surgery.

The red arrow indicates the repositioned mandibular component of the TMJ prosthesis. The red lines indicate the approximately aligned maxillary and mandibular dental midlines.
