# Supplementary material for: Management of recurrent posterior dislocation of a stock temporomandibular joint prosthesis using class II elastic traction: A case report and literature review
Source: Medicine (Baltimore). 2026 Oct 2;105(40):e50718. doi: 10.1097/MD.0000000000050718 (PMC13633035; doi:10.1097/MD.0000000000050718)
Supplement: Supplementary file 4 [file medi-105-e50718-s004.pdf]

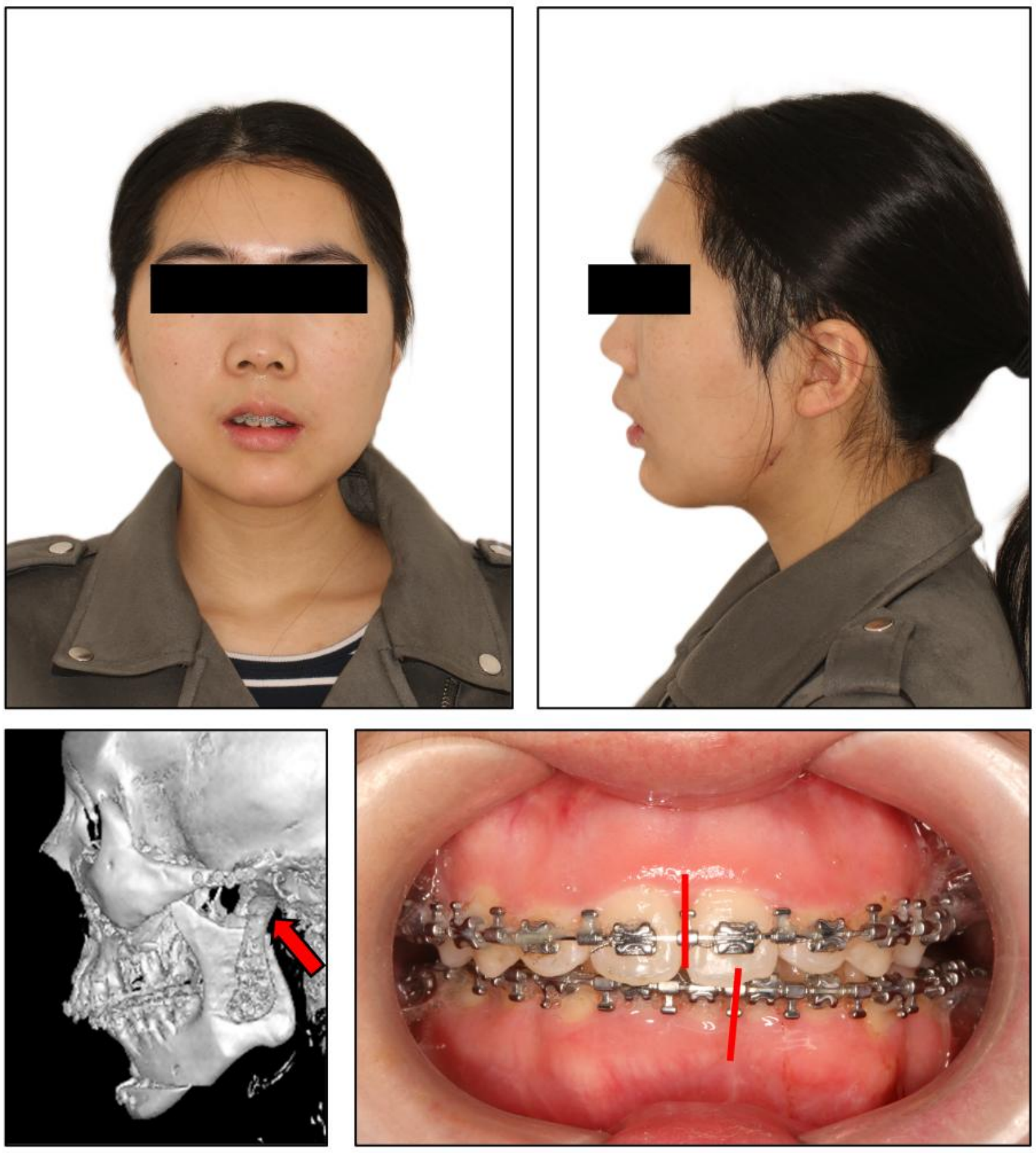

Supplementary figure 4. Facial/CT images and intraoral photograph 37 days after discharged.

The red arrow indicates the recurrently posteriorly dislocated mandibular component of the TMJ prosthesis. The red lines mark the dental midlines, showing leftward deviation of the mandibular midline.
