# Supplementary material for: Management of recurrent posterior dislocation of a stock temporomandibular joint prosthesis using class II elastic traction: A case report and literature review
Source: Medicine (Baltimore). 2026 Oct 2;105(40):e50718. doi: 10.1097/MD.0000000000050718 (PMC13633035; doi:10.1097/MD.0000000000050718)
Supplement: Supplementary file 5 [file medi-105-e50718-s005.pdf]

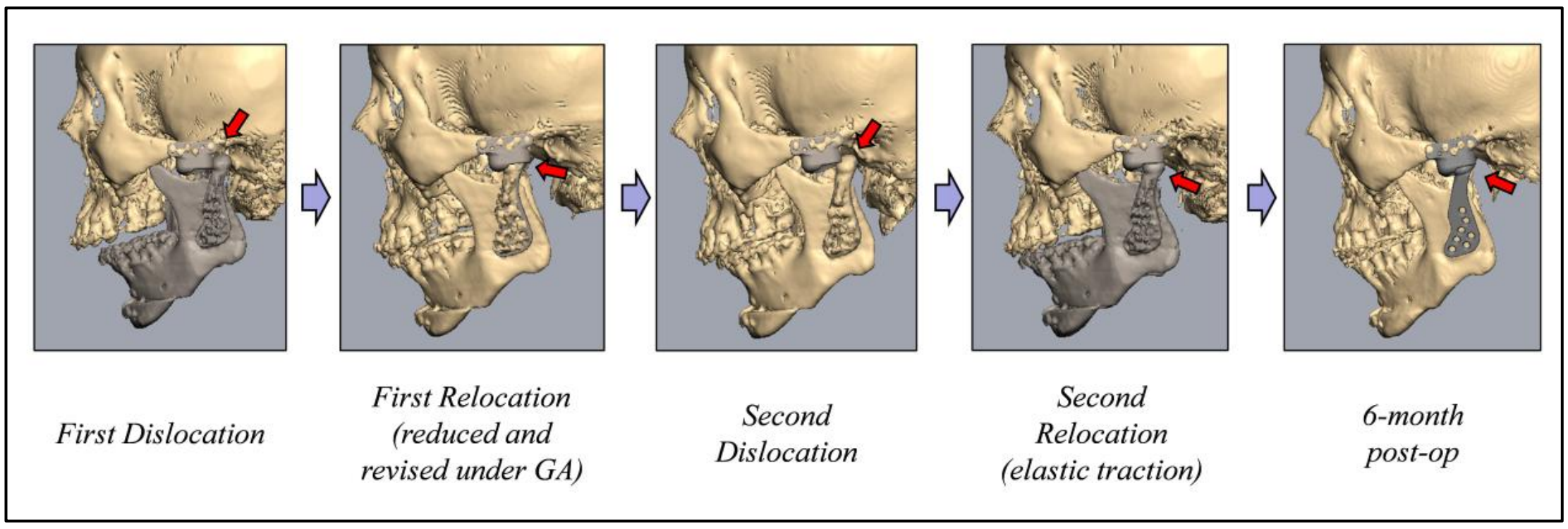

Supplementary figure 5. Reconstruction of the joint and fossa prostheses throughout treatment.

The red arrows indicate the mandibular components of the TMJ prostheses. The reconstructed fossa prostheses were registered to their actual positions using the screw holes as landmarks, enabling visualization of the spatial relationship between the mandibular and fossa components at each time point.
